# Convolutional Neural Network-Processed MRI Images in the Diagnosis of Plastic Bronchitis in Children

**DOI:** 10.1155/2021/2748830

**Published:** 2021-09-13

**Authors:** Xiaoqun Chen, Rong Lu, Feng Zhao

**Affiliations:** ^1^Department of Pediatric Internal Medicine, Northwest Women's and Children's Hospital, Xi'an, Shaanxi 710061, China; ^2^Department of Pediatrics, Affiliated Hospital of Yan'an University, Yan'an 716000, Shaanxi, China

## Abstract

**Objective:**

The study focused on the features of the convolutional neural networks- (CNN-) processed magnetic resonance imaging (MRI) images for plastic bronchitis (PB) in children.

**Methods:**

30 PB children were selected as subjects, including 19 boys and 11 girls. They all received the MRI examination for the chest. Then, a CNN-based algorithm was constructed and compared with Active Appearance Model (AAM) algorithm for segmentation effects of MRI images in 30 PB children, factoring into occurring simultaneously than (OST), Dice, and Jaccard coefficient.

**Results:**

The maximum Dice coefficient of CNN algorithm reached 0.946, while that of active AAM was 0.843, and the Jaccard coefficient of CNN algorithm was also higher (0.894 vs. 0.758, *P* < 0.05). The MRI images showed pulmonary inflammation in all subjects. Of 30 patients, 14 (46.66%) had complicated pulmonary atelectasis, 9 (30%) had the complicated pleural effusion, 3 (10%) had pneumothorax, 2 (6.67%) had complicated mediastinal emphysema, and 2 (6.67%) had complicated pneumopericardium. Also, of 30 patients, 19 (63.33%) had lung consolidation and atelectasis in a single lung lobe and 11 (36.67%) in both two lung lobes.

**Conclusion:**

The algorithm based on CNN can significantly improve the segmentation accuracy of MRI images for plastic bronchitis in children. The pleural effusion was a dangerous factor for the occurrence and development of PB.

## 1. Introduction

Plastic bronchitis (PB) is a rare respiratory disease arising from pathogenic infections. The endogenous foreign matter produced then leads to bronchial congestion, ventilatory disorder, and air exchange dysfunction [1]. Clinically, it manifests as dyspnea, wheezing, chest pain, fever, and even life-threatening respiratory circulatory failure in severe cases. Children aged 4–12 are predominantly affected [2]. According to the pathological characteristics, it mainly falls into type I and type II plastic bronchitis. Type I refers to the inflammatory type, where fibrous protein and a great number of inflammatory cells are visible in the bronchus, thanks to infectious diseases of the respiratory tract. Type II is cell-free, where the bronchus is mainly blocked with mucoprotein, without or with just a few infiltration cells, and a great amount of lymph fluid is visible in the alveolus [3]. The incidence of PB is increasing in China, and it is associated with the *Mycoplasma pneumoniae* infection or virus infection of the respiratory tract [4].

Electronic computed tomography (CT) is a routine examination to diagnose plastic bronchitis in children. With the continuous maturity of CT technology, CT has gradually become the first choice for lung examination and is a common examination method to determine various diseases. However, the effective radiation dose involved in CT examination is relatively harmful to children, and CT examination can only observe the macroscopic signs of the lesion site, unable to accurately determine the shape, size, and density, and the conclusion is relatively simple, which has certain limitations in the diagnosis of plastic bronchitis in children. Magnetic resonance imaging (MRI) belongs to positron emission tomography (PET), during which the radio-frequency pulse at a specific frequency is applied to the human body in a magnetostatic field, to motivate the hydrogen proton in the human body, triggering magnetic resonance. Next, the electromagnetic signal of the human body is used to reconstruct the image [5]. MRI mainly consists of three systems, namely, the magnet system, the spectrometer system, and the computer image reconstruction system [6, 7]. During the examination, the patient is in a magnetic field and the orientation of magnetic moment is consistent with the direction of magnetic induction line. The radio-frequency pulses enable the lower-energy-level nuclei to jump to the higher-energy level by absorbing the radio-frequency energy and disrupting the proton movement in the tissue. When the pulses are stopped, the energy level and phase position of protons return to the premotivated state. This process is called the relaxation [8]. T1-weighted image (T1WI) can display the anatomical details, while T2-weighted image (T2WI) can display lesions. The black-white contrast of MRI stems from the signal differences in different tissues. Short T1 presents white, and long T1 presents black; long T2 presents white, and short T2 presents black. The air and bone cortex presents black no matter in T1 or T2. The infarction, inflammation, tumor, and fluid present low-signal T1WI but high-signal T2WI [9]. MRI can display the anatomical structure of any section of the human body, with high resolution. It can clearly display the lesions in the spinal cord, brainstem, fossa cranii posterior, and bronchi. Additionally, MRI is free of ionizing radiation and carcinogenic risk. It is a very safe examination. However, MRI examination also has certain limitations, and image artifacts are often produced by the influence of respiration and blood flow, which hinder the application of MRI in lung imaging. High-quality images are beneficial to the diagnosis of lung diseases, which can fully reflect the details of the image and significantly improve the diagnostic coincidence rate.

The convolutional neural network (CNN) is a feedforward neural network. It has a deep structure and contains convolutional calculation and is representative of deep learning [10]. In the medical field, the CNN is usually used to learn original MRI images and then applied in image segmentation and image classification. Sathish et al. (2019) [11] put forward in their study that the pixel information of different dimensions can be combined to extract the optimal size information. Wang et al. (2019) [12] believed that reducing the size of convolution kernels can improve the operating speed of the neural networks.

In the study, the CNN-based algorithm and Active Appearance Model- (AAM-) based algorithm were compared for the segmentation results of MRI images in 30 PB children. The objective of the study was to explore the features of MRI images for PB patients, expected to provide reliable reference for its clinical diagnosis.

## 2. Materials and Methods

### 2.1. Subjects and Grouping

In the study, 30 PB patients diagnosed in xx Hospital from Jan. 2019 to Dec. 2020 were selected as the subjects, including 19 boys and 11 girls, and they all underwent the MRI examination. They were between 6 and 12 years old, with an average age of 8.21 ± 1.25 years old. The study was approved by the Ethics Committee of xx Hospital, and all the subjects had signed an informed consent form.

Inclusion criteria were as follows: (1) children diagnosed with plastic bronchitis by hospital pathological examination; (2) children with good coordination during the examination; (3) children with complete general data and imaging data.

Exclusion criteria were as follows: (1) children with suspected clinical diagnosis of plastic bronchitis and no plastic material found in imaging examination; (2) children who had contraindications; (3) children with mental diseases; (4) children who could not normally cooperate with the examination.

### 2.2. Structure of the CNN Model

**Figure 1:**
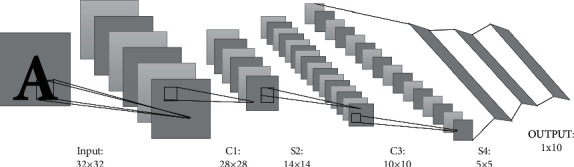
Structure of the CNN model.

Figure 1 shows the structure of the CNN model. LeNet-5 is used in the study. The input data include a matrix formed by 32 × 32 pixels. The first feature layer includes 6 feature maps. After a 5 × 5 window is used to convolve the input image, a 28 × 28 feature map can be obtained. Then, downsampling is performed on the first feature layer to obtain 6 feature maps with a size of 14 × 14. C stands for the convolution layer, and S stands for the pooling layer. The C3 layer is a convolutional layer, and the size of the convolution kernel is 5 × 5, as with C1. The downsampling is performed again in S4. The C5 layer performs a convolution operation on the S4 layer, and each convolution kernel in C5 layer is convolved on the basis of the S4 feature map. Then, on the basis of the C5 layer, through a fully connected network, a result of 1 × 10 is finally obtained as output. In the vector of the output result of 1 × 10, the image of the final output model is classified by ReLu function and the classification result of the network output is the corresponding position of the maximum component.

### 2.3. CNN-Based PB Segmentation Model

The convolutional neural network is mainly composed of the convolutional layer, the pooling layer, the fully connected layer, and the deconvolution layer. The convolutional layer detects the local features of MRI images, which can be expressed as follows:

(1) $$\begin{array}{c} B_{j}^{l}=g\left(\sum_{i=1}^{M\left(l-1\right)}B_{i}^{l-1}•k_{ij}^{l}+c_{j}^{l}\right), \end{array}$$

where *k*_*ij*_^*l*^ represents the convolution kernel, *i* indicates the feature map in the *l* − 1 layer, *j* represents the feature map in the *l* layer, *B*_*j*_^*l*^ indicates the feature map in the convolutional layer, and *B*_*i*_^*l*−1^ represents the feature map adjacent to *B*_*j*_^*l*^. *M*(*l* − 1) indicates the number of feature maps in the *l* − 1 layer, • indicates the convolution, *c*_*j*_^*l*^ is the bias, and *f*() represents the nonlinear activation function.

The activation function is to process linear nonseparable data. The study uses ReLu as the activation function, expressed as Equation (2), and the relevant derivative function can be expressed in Equation (3).

(2) $$\begin{array}{c} g\left(x\right)=x^{+}=\max\left(0,x\right), \end{array}$$

(3) $$\begin{array}{c} g^{\prime }\left(x\right)=\left\{\begin{array}{cc} x & x>0,\\ 0 & x\leq 0. \end{array}\right.. \end{array}$$

As for an MRI image A, *X* is the set of all pixels in image A: *X*=(*x*_1_, *x*_2_,…, *x*_*n*_) and B indicates segmentation categories (*b*_1_, *b*_2_,…, *b*_*m*−1_), where *m*=4; the probability to output *b*_*j*_ of pixel *x*_*i*_ in the *j* channel is expressed as follows:

(4) $$\begin{array}{c} p\left(x_{i}=b_{j}\right)=\frac{1}{Z}\exp\left[v\left(b_{j}\right)\right], \end{array}$$

where *v*(*b*_*j*_) indicates the value of *b*_*j*_, *Z* is the regularized item, the predicted value *y*_*i*_ of *x*_*i*_ is expressed as *y*_*i*_=argmax[*p*(*x*_*i*_=*b*_*j*_)], and the relevant loss function is expressed as follows:

(5) $$\begin{array}{c} \text{loss}=-\frac{1}{mn}\sum_{i}\sum_{j}y_{ij}\ln\left[p\left(x_{i}=b_{j}\right)\right]. \end{array}$$

Adam is a commonly used method to optimize neural networks, and it is employed in the study to optimize the CNN model. The update parameter *W* and the offsets *S*_*dw*_ and *V*_*dw*_ can be expressed as follows:

(6) $$\begin{array}{c} V_{dw}=\alpha V_{dw}+\left(1-\alpha \right)\frac{\partial P}{\partial W}, \end{array}$$

(7) $$\begin{array}{c} S_{dw}=\alpha_{2}S_{dw}+\left(1-\alpha_{2}\right){\left(\frac{\partial P}{\partial W}\right)}^{2}, \end{array}$$

(8) $$\begin{array}{c} W=W-\beta \frac{V_{dw}}{\sqrt{S_{dw}}+\varepsilon }, \end{array}$$

where *P* is the circulation number of Adam, *α*_1_ and *α*_2_ indicate the hyperparameters to control average weighted value of the two coefficients, *β* is the learning efficiency, and *ε* is a small constant, which is set to avoid that the denominator is zero. The parameters of the convolutional neural network are shown in Table 1.

### 2.4. MRI Images Processed by the CNN-Based Model

The experimental equipment is a workstation equipped with Quadro K40c graphics card, 128 G memory, Intel Xeon(R) CPU E5-2660 V3, and Ubuntu 14.04 LTS (64 bit) system. The entire training is completed under the Tensorflow framework. The testing phase can be completed either in the MATLAB 2015B platform or in the Tensorflow framework.

Occurring simultaneously than (OST) factor is used to evaluate the quality of MRI image. It is the area of the intersection to the area of the union. A higher OST indicates a more accurate target detection bounding box.

It is calculated as follows:

(9) $$\begin{array}{c} OST=\frac{\text{area}\left(S\right)\cap \text{area}\left(P\right)}{\text{area}\left(S\right)\cup \text{area}\left(P\right)}, \end{array}$$

where *S* represents the standard area segmented by the doctor and *P*represents the result segmented by the CNN model. The shape of the bronchus is mostly variable and irregular, so the Dice coefficient, precision, and Jaccard coefficient were used to evaluate the segmentation effects. The Dice coefficient is a measure of the overlap between the segmentation result and the gold standard area. It is calculated as follows:

(10) $$\begin{array}{c} \text{Dice}\left(S,P\right)=2\times \frac{S\cap P}{S+P}=2\frac{TPV}{TPV+FPV+TPV+FN}, \end{array}$$

where *S* represents the standard area segmented by the doctor and *P*represents the result segmented by the CNN model. *TPV*(true positive) means that the segmentation result and the gold standard result are both true; *P* means that the segmentation result is false and the gold standard result is true; and FN (false negative) means that the segmentation result and the gold standard result are both false. A smaller Dice coefficient indicates a larger gap between the predicted result and the real result.

The precision is calculated as follows:

(11) $$\begin{array}{c} \text{accuracy}=\frac{TPV}{TPV+FN}\times 100\%. \end{array}$$

The Jaccard coefficient reflects the difference between the data, and it is calculated as follows:

(12) $$\begin{array}{c} \text{Jaccard}=\frac{\left|E\cap F\right|}{\left|E\cup F\right|}\times 100\% \end{array}$$

where *E* is the left bronchus predicted by the CNN and *F* represents the right bronchus area delineated by the doctor.

### 2.5. MRI Scanning Method

MR Prisma 3.0 (SIEMENS, Germany) is used to conduct the scanning of 30 patients. During the scanning, the child lied on his/her back and was instructed to breathe steadily. For the child who did not cooperate, taking chloral hydrate orally was required and the doctor should inject chlorpromazine and Phenergan intramuscularly to sedate him/her. During examination, it is a must to ensure that the child maintains one posture all the time. First, cross-section and sagittal-section scans were performed to find the tracheal plane. Then, coronal-section scan was performed with the point lightly above the crotch of the trachea and bronchus as the central point. Scanning parameters were as follows: matrix is 251 × 251; layer thickness is 3.5 mm; vision is 25 × 25 cm; the flip angle is 15°; and the slice gap is 6.1 mm. After the scan, two experienced professional physicians in the imaging department performed manual segmentation and analysis of the image.

### 2.6. Statistical Method

The data were processed by SPSS20.0. The measurement data were expressed as mean ± deviation ($\bar{x}$ ± *s*), and *t*-test was adopted. The count data were expressed by percentage, and *χ*^2^ test was used. *P* < 0.05 was the threshold for significance.

## 3. Results

### 3.1. Analysis of Bronchial Detection Results Based on Convolutional Neural Network Algorithm

**Figure 2:**
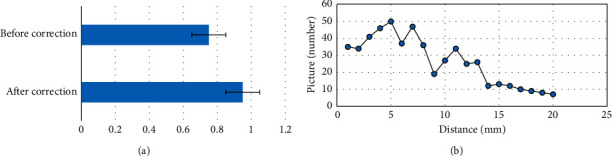
Detection results of the bronchus. (a) OST value before and after correction. (b) Euclidean distance.

Figure 2(a) shows the OST values before and after correction. Before correction, the mean OST value of target box and real box was 0.658. After correction, it was 0.921, increasing by 0.263.  Figure 2(b) shows the Euclidean distance between the central point predicted by the CNN-based model and the real central point. It was between 0 and 20 mm and mainly ranged from 4 to 10 mm. Further statistics showed that the Euclidean distance less than 10 mm accounted for 72% among those ranging from 0 to 20 mm.

### 3.2. Evaluation of MRI Image Quality of Children Based on Convolutional Neural Network Processing

**Figure 3:**
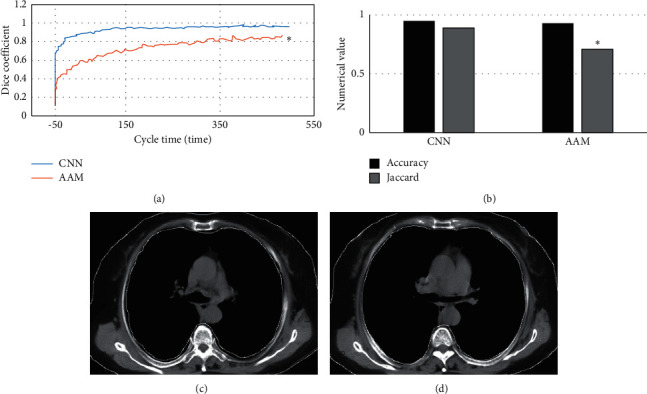
The CNN-processed MRI image. (a) Dice coefficient of two algorithms in different cycles. (b) Precision and Jaccard coefficient of the two algorithms. (c) Segmentation result of the CNN model. (d) Segmentation result of the AAM model. ^*∗*^A statistically significant difference versus CNN, *P* < 0.05.

AAM is an image feature extraction method, which can be used to extract features from target images.  Figure 3(a) shows the Dice coefficients of CNN-processed MRI images and AAM-processed MRI images under different training cycles. As the number of training cycles increased, the Dice coefficients of both the two algorithms rose, but the Dice coefficient of CNN-based algorithm was always higher than that of AAM model. The maximum Dice coefficient of CNN-based algorithm reached 0.946, while that of AAM was 0.843. It indicated that the results predicted by the CNN model had smaller differences from the real results versus the AAM.

Figure 3(b) shows the precision and Jaccard coefficient under the two algorithms compared. The precision of CNN algorithm was 0.952, while that of the AAM model was 0.934, showing no obvious difference (*P* > 0.05). The Jaccard coefficient of CNN algorithm was 0.894, while that of the AAM model was 0.758, and the difference was statistically significant (*P* < 0.05). Figures 3(c) and 3(d) show the segmentation results of the CNN model and AAM model, respectively.

### 3.3. General Statistics on Pediatric Patients

**Figure 4:**
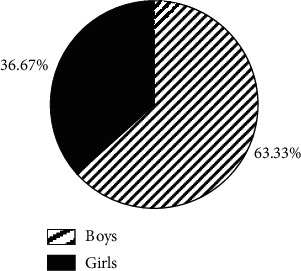
Gender distribution of PB patients.

**Figure 5:**
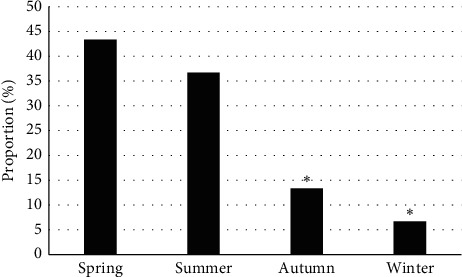
Distribution of onset time. ^*∗*^A statistically significant difference versus spring, *P* < 0.05.

**Figure 6:**
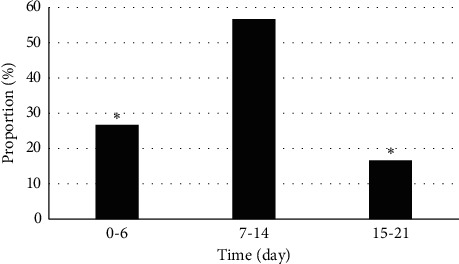
Hospital stay of PB patients. ^*∗*^A statistically significant difference versus the hospital stay of 7–14 days, *P* < 0.05.

Of 30 PB patients, the boys (19) outnumbered the girls (11) by a wider margin and the difference was statistically significant (*P* < 0.05) (Figure 4). The subjects aged from 6 to 12 years, with an average age of 8.21 ± 1.25 years. The onset time of PB was concentrated in spring and summer, and there were 13 cases in spring (43.33%), 11 cases in summer (36.67%), 4 cases in autumn (13.33%), and 2 cases in winter (6.67%). Obviously, the incidence in autumn and winter was lower than in spring (*P* < 0.05) (Figure 5). The average hospital stay was 13.2 ± 7.6 days. Of the 30 patients, 17 had a hospital stay of 7–14 days (56.67%), 8 had a hospital stay of 0–6 days (26.67%), and 5 had a hospital stay of 15–21 days (16.66%) (Figure 6).

### 3.4. MRI Images Features Processed by CNN-Based Algorithm

**Figure 7:**
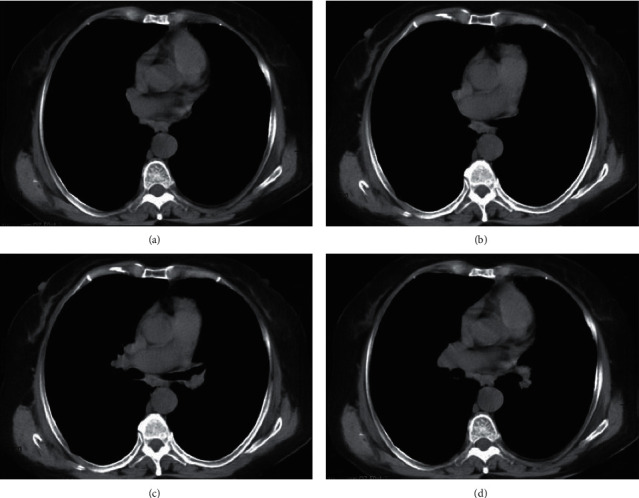
MRI images for PB patients. (a) MRI image of a 7-year-old male patient with chronic emphysema and pleural thickening and adhesion. (b) MRI image of a 10-year-old child with aortic dissection. (c) MRI image of an 11-year-old child with pleural calcification near the right upper mediastinum. (d) MRI image of a 10-year-old male child with double-cavity signs.

Figure 7 shows MRI images processed by CNN-based algorithm.  Figure 7(a) presents chronic emphysema and pleural thickening and adhesions; Figure 7(b) presents aortic dissection; Figure 7(c) presents calcification of the pleura beside the right upper mediastinum; and Figure 7(d) presents the double cavities. The MRI images clearly display the trend of the trachea and bronchus, location, morphology, size, and density of foreign matters. It manifested that the solidified sticky secretion was tubular in shape and its density was close to or slightly higher than the density of liquid. Furthermore, there were high-density dot or strip shadows.

### 3.5. MRI Image Manifestations of PB Patients

**Figure 8:**
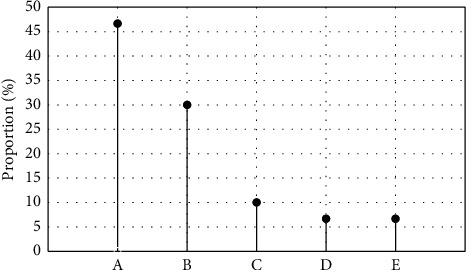
MRI imaging manifestations of PB patients. (a) Pulmonary atelectasis. (b) Pleural effusion. (c) Mediastinal emphysema. (d) Pneumothorax. (e) Pneumopericardium.

**Figure 9:**
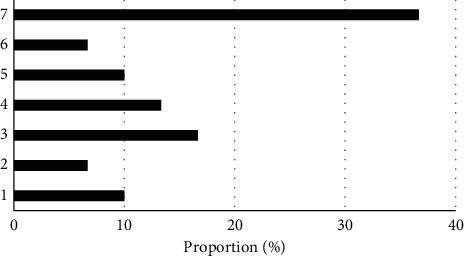
Distribution of lung consolidation and atelectasis. 1: left upper lobe; 2: left middle lobe; 3: left lower lobe; 4: right upper lobe; 5: right middle lobe; 6: right lower lobe; 7: bilateral lobes.

The pulmonary inflammation was visible in all subjects. Of 30 PB patients, 14 (46.66%) had complicated pulmonary atelectasis, 9 (30%) had complicated pleural effusion, 3 (10%) had pneumothorax, 2 (6.67%) had complicated mediastinal emphysema, and 2 (6.67%) had complicated pneumopericardium (Figure 8). Also, of 30 patients, 19 (63.33%) had lung consolidation and atelectasis in a single lung lobe and 11 (36.67%) in both two lung lobes (Figure 9).

## 4. Discussion

PB arises from the block of bronchus, leading to abnormal ventilation of the lung tissue and even acute dyspnea in severe cases. Generally, foreign matters are in a tree-like shape [13]. PB may occur at any stages, but children are predominantly affected, especially those aged 4–12 years [14]. The PB subjects in the study were from 6 to 12 years old, with an average age of 8.21 ± 1.25 years. Of 30 PB patients, boys outnumbered girls and the difference was statistically significant (*P* < 0.05), which was consistent with the research results of Xiong et al. (2019) [15]. The outset time was concentrated in spring and summer, suggesting that the onset of PB is seasonal.

Before correction, the average OST value of target box and real box was 0.658. After correction, it was 0.921. The Euclidean distance from the predicted central point to the real central point ranged from 0 to 20 mm and the Euclidean distance less than 10 mm accounted for 72.5%. It indicated that the CNN-based algorithm can accurately segment MRI images for PB. The Dice coefficient has been widely applied in the verification of the segmentation effects [16]. Under different number of training cycles, the maximum Dice coefficient of CNN-based algorithm reached 0.946, while that of AAM algorithm was 0.843. It indicated that the algorithm of the study had better segmentation effects, which was in line with the research results of Fujioka et al. (2021) [17].

Clinically, PB mainly manifests as fever and cough [18, 19]. A majority of subjects included had fever. The average hospital stay was 13.2 ± 7.6 days. Of the 30 patients, 17 had a hospital stay of 7–14 days (56.67%), 8 had a hospital stay of 0–6 days (26.67%), and 5 had a hospital stay of 15–21 days (16.66%). The MRI images for PB presented chronic emphysema, pleural thickening and adhesions, aortic dissection, calcification of the pleura beside the right upper mediastinum, and double-cavity signs. MRI can clearly display the trend of the trachea and bronchus, location, morphology, size, and density of foreign matters. It manifested that the solidified sticky secretion was tubular in shape and its density was close to or slightly higher than the density of liquid. Furthermore, there were high-density dot or strip shadows.

Of 30 patients, 19 (63.33%) had lung consolidation and atelectasis in a single lung lobe and 11 (36.67%) in both two lung lobes, which was aligned with the research results of Lee et al. (2018) [20]. Also, of 30 PB patients, 14 (46.66%) had complicated pulmonary atelectasis, 9 (30%) had complicated pleural effusion, 3 (10%) had pneumothorax, 2 (6.67%) had complicated mediastinal emphysema, and 2 (6.67%) had complicated pneumopericardium. It prompted that the pleural effusion may be a dangerous factor in the occurrence and development of PB.

## 5. Conclusion

In this study, a CNN-based algorithm was constructed and applied to segment MRI images for 30 PB patients, to explore the imaging features of PB patients. The algorithm based on CNN can significantly improve the segmentation accuracy of MRI images for plastic bronchitis in children. MRI images prompted that pleural effusion was a dangerous factor in the occurrence and development of PB. However, the shortcomings of this study are that the sample size is small, the MRI examination time is long, and the cost is high, which is limited in the actual operation. In short, the CNN-based MRI image segmentation model greatly raises the segmentation accuracy of the bronchus, providing a theoretical basis for the diagnosis of PB.

## Figures and Tables

**Table 1:** Convolution neural network parameter configuration table.

Category	Size	Number	Step size	Fill
Conv1	5	512	1	2
Pooling1	3	512	1	0
Conv2	5	384	1	1
Pooling2	3	384	2	0
Conv3	3	256	2	0
Pooling3	3	256	1	0
Fully connected 5	1	9216	2	0

## Data Availability

The data used to support the findings of this study are available from the corresponding author upon request.
